# Prediction of hepatitis E using machine learning models

**DOI:** 10.1371/journal.pone.0237750

**Published:** 2020-09-17

**Authors:** Yanhui Guo, Yi Feng, Fuli Qu, Li Zhang, Bingyu Yan, Jingjing Lv

**Affiliations:** 1 School of Data and Computer Science, Shandong Women’s Unversity, Jinan, Shandong, China; 2 Shandong Provincial Key Laboratory of Infectious Disease Control and Prevention, Shandong Center for Disease Control and Prevention, Jinan, Shandong, China; 3 Academy of Preventive Medicine, Shandong University, Jinan, Shandong, China; Polytechnical Universidad de Madrid, SPAIN

## Abstract

**Background:**

Accurate and reliable predictions of infectious disease can be valuable to public health organizations that plan interventions to decrease or prevent disease transmission. A great variety of models have been developed for this task. However, for different data series, the performance of these models varies. Hepatitis E, as an acute liver disease, has been a major public health problem. Which model is more appropriate for predicting the incidence of hepatitis E? In this paper, three different methods are used and the performance of the three methods is compared.

**Methods:**

Autoregressive integrated moving average(ARIMA), support vector machine(SVM) and long short-term memory(LSTM) recurrent neural network were adopted and compared. ARIMA was implemented by python with the help of statsmodels. SVM was accomplished by matlab with libSVM library. LSTM was designed by ourselves with Keras, a deep learning library. To tackle the problem of overfitting caused by limited training samples, we adopted dropout and regularization strategies in our LSTM model. Experimental data were obtained from the monthly incidence and cases number of hepatitis E from January 2005 to December 2017 in Shandong province, China. We selected data from July 2015 to December 2017 to validate the models, and the rest was taken as training set. Three metrics were applied to compare the performance of models, including root mean square error(RMSE), mean absolute percentage error(MAPE) and mean absolute error(MAE).

**Results:**

By analyzing data, we took *ARIMA*(1, 1, 1), *ARIMA*(3, 1, 2) as monthly incidence prediction model and cases number prediction model, respectively. Cross-validation and grid search were used to optimize parameters of SVM. Penalty coefficient *C* and kernel function parameter *g* were set 8, 0.125 for incidence prediction, and 22, 0.01 for cases number prediction. LSTM has 4 nodes. Dropout and L2 regularization parameters were set 0.15, 0.001, respectively. By the metrics of RMSE, we obtained 0.022, 0.0204, 0.01 for incidence prediction, using ARIMA, SVM and LSTM. And we obtained 22.25, 20.0368, 11.75 for cases number prediction, using three models. For MAPE metrics, the results were 23.5%, 21.7%, 15.08%, and 23.6%, 21.44%, 13.6%, for incidence prediction and cases number prediction, respectively. For MAE metrics, the results were 0.018, 0.0167, 0.011 and 18.003, 16.5815, 9.984, for incidence prediction and cases number prediction, respectively.

**Conclusions:**

Comparing ARIMA, SVM and LSTM, we found that nonlinear models(SVM, LSTM) outperform linear models(ARIMA). LSTM obtained the best performance in all three metrics of RSME, MAPE, MAE. Hence, LSTM is the most suitable for predicting hepatitis E monthly incidence and cases number.

## Introduction

Viral hepatitis is recognized as one of the most frequently reported diseases, and hepatitis E as an acute liver disease has been a major public health problem [1]. Every year, there are an estimated 20 million hepatitis E infections worldwide, leading to over 3 million symptomatic cases of hepatitis E, and 55,000 hepatitis E-related deaths. The prevalence is highest in East and South Asia [2]. Sporadic hepatitis E has caused over 50% of acute viral hepatitis cases in recent years [3], which caused the huge social, economic, and health burden. However, incidence data relying on hospital-based reporting, is often lagged. To better mitigate future outbreaks, it is necessary to accurately predict the incidence of hepatitis E. US Centers for Disease Control (CDC) and Prevention have openly endorsed adopting models to inform decision making [4].

Hepatitis E is transmitted by the fecal-oral route through contaminated water. It mainly broke out in developing countries in Asia, Africa and Central America [5]. In recent years, the incidence and death of hepatitis E are higher than that of hepatitis A, and the incidence is on the rise. With the development of information technology, CDC has accumulated a large number of historical data of hepatitis E. Effective use of these data to predict the incidence can reduce the risk of hepatitis E. However, researches on prediction and early warning of infectious diseases mainly focus on dengue [6, 7], influenza [8], AIDS [9], and hepatitis B [10, 11]. There are few studies on hepatitis E incidence. Hence, this paper focuses on the key issue of hepatitis E incidence.

For the prediction of infectious diseases, researchers mainly adopt time series method. Originally, some linear estimation methods were applied to the prediction of infectious diseases, including Autoregressive(AR) [12], Moving Average(MA) [13] and Autoregressive Moving Average(ARMA) [14]. However, the above methods are only applicable to stationary data. Subsequently, ARIMA model was proposed, which is better to address data with some trend. The paper [11] adopted ARIMA to predict incidence of hepatitis B. They showed that ARIMA model outperformed grey model GM(1,1). An improved ARIMA model, called SARIMA, which takes into account recent and seasonal patterns, has been shown to produce useful disease estimates. The paper [15] adopted SARIMA model to capture a substantial amount of dengue variability, and obtained better results.

Besides, another mainstream to analyze time series is adopted by artificial intelligence methods, such as Markov model [16], artificial neural network [17], support vector machine(SVM) [18], etc. Because of the generalization ability and the ability to process high-dimensional nonlinear regression estimation, SVM has been successfully used in many fields of time series prediction, including financial prediction [19] and disease prediction [20]. The paper [20] applied SVM to predict dengue incidence, obtained better results.

At present, deep learning represented by Convolutional Neural Network(CNN) [21] and Recurrent Neural Network(RNN) [22], has revolutionized various fields, due to the powerful feature extraction and representation capabilities. Among them, CNN is widely used in image recognition, classification and other visual problems. RNN is a powerful approach to analyze temporal data, and widely used in natural language processing [22], speech recognition [23] and so on. However, when there are many recursions, RNN fails in practice due to problems with vanishing gradients [24]. Moreover, RNN can not satisfy the multimodal case because of sharing parameters. Fortunately, Long Short-Term Memory(LSTM), a variant of the RNN, makes up for the lack of RNN. Nowadays, LSTM has been used in various fields, including traffic flow prediction [25], finacial prediction [26], infectious diseases prediction [27]. The paper [27] applied LSTM to model seasonality and trends in hand-foot-mouth disease incidence, and got a good result.

In this paper, ARIMA, SVM and LSTM were used to predict the monthly incidence of hepatitis E in Shandong Province. We use RMSE, MAPE and MAE to evaluate the three methods. Specially, LSTM model obtained state-of-the-art performance. The model building and comparison would give some suggestions on the model chosen. And the predicted results may offer references for hepatitis E prevention. Meanwhile, these methods are general and could also be suitable for predicting other diseases.

## Materials and methods

### Materials source

**Fig 1:**
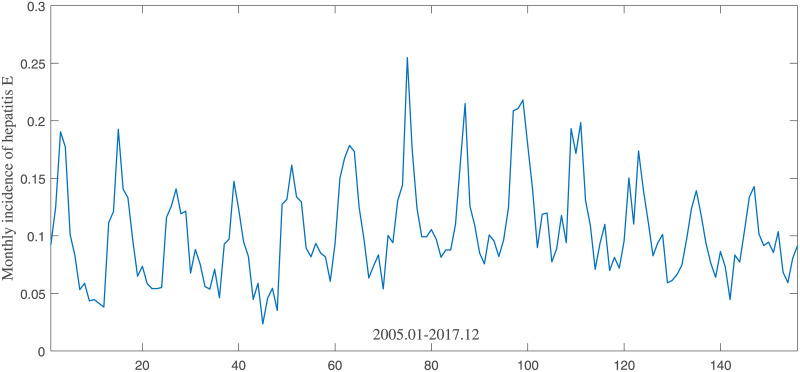
Monthly incidence of hepatitis E from January 2005 to December 2017.

**Fig 2:**
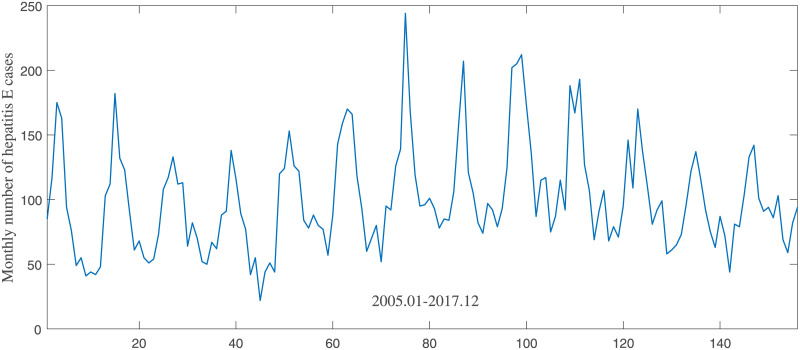
Monthly cases number of hepatitis E from January 2005 to December 2017.

We obtained publicly available data about hepatitis E in Shandong Province, China between 2005 and 2017 from the Shandong Center for Disease Control and Prevention(SCDC). Data mainly includes monthly incidence and monthly cases number of hepatitis E in Shandong. Monthly incidence means the number of per 100,000 people in Shandong Province, as shown in Fig 1. Monthly cases number is the number of aggregated confirmed cases in a month, as shown in Fig 2.

### ARIMA model

ARIMA model consists of auto regressive (AR) model and moving average (MA) model. The model is expressed as *ARIMA*(*p*, *d*, *q*), in which *p* is the order of auto regression, *d* is the degree of trend difference, *q* means the order of moving average. First, we can determine the parameter *d* by evaluating the stationarity of the data. Then, we determine *p* and *q* by analyzing autocorrelation and partial correlation. Finally, training and prediction are done.

#### Data stationarity analysis

In the analysis of time series, the basic assumption is the stationarity and ergodicity of the series. An important tool of testing time series stationarity is the Augmented Dickey-Fuller(ADF) unit root test. If the data series is not stationary, we need to adopt some transformation methods to make the data stationary, including logarithmic transform, smoothing methods, difference methods and decomposition methods. In this paper, difference method is used to meet the requirement of ARIMA model.

#### Parameter estimation

After stationarity analysis, we can determine parameter *d*. *p* and *q* of ARIMA model are estimated by autocorrelation function(ACF) and partial autocorrelation function(PACF). In order to get a more efficient ARIMA model, we optimize the parameters *p* and *q* by the grid-search method, according BIC criterion. Finally, we choose *ARIMA*(1, 1, 1) and *ARIMA*(3, 1, 2) for incidence and cases number prediction of hepatitis E, respectively.

### SVM model

SVM was proposed by Vapnik [28], widely applied to solve classification and regression problems. SVM is more suitable for nonlinear problem by kernel function, and improves the generalization ability of model by structural risk. SVM regression also is called SVR, for regression problem. In this work, we use libSVM (http://www.csie.ntu.edu.tw/~cjlin/libsvm) designed by Lin [29] to implement hepatitis E incidence prediction.

#### Data preprocessing and modeling

Firstly, we normalize the raw data to [0, 1] by min-max normalization. The normalization formula is *x*_*norm*_ = (*x* − *x*_*min*_)/(*x*_*max*_ − *x*_*min*_), where *x* denotes the raw data, *x*_*min*_ and *x*_*max*_ are minimum and maximum values, respectively. By observing the autocorrelation of hepatitis data, we use previous three data to predict the next one, *x*_*t*_ = *f*(*x*_*t*−3_, *x*_*t*−2_, *x*_*t*−1_). We choose 80% of the data as the training set and the rest as the test set.

#### Parameters setting of SVM

The kernel function, as the similarity metrics of the samples, is a key factor that affects SVM model. We adopted radial basis function(RBF) as kernel function. In addition, penalty coefficient *C* and *g* are also important parameters that affect the performance of SVM. We use grid searching to find the optimal combination of parameters. *C* and *g* change from 2^−10^ to 2^5^, by index changes. In our experiment of hepatitis E incidence, *C* and *g* were set by 8, 0.125, respectively. For the prediction of hepatitis E cases number, *C* and *g* were set by 22, 0.01, respectively.

### LSTM model

LSTM is variant of RNN, which can deal with long-term sequential data since the gradients tend to vanish. LSTM’s ability is mainly due to the existence of memory unit, usually referred to as cell state. Cell state can determine whether the information is useful. Then, it save the useful information. There are three gates in a cell, which are called input gate, forget gate and output gate. In this paper, we implemented LSTM with the help of Keras (https://pypi.org/project/Keras/).

#### Data preprocessing and modeling

**Fig 3:**
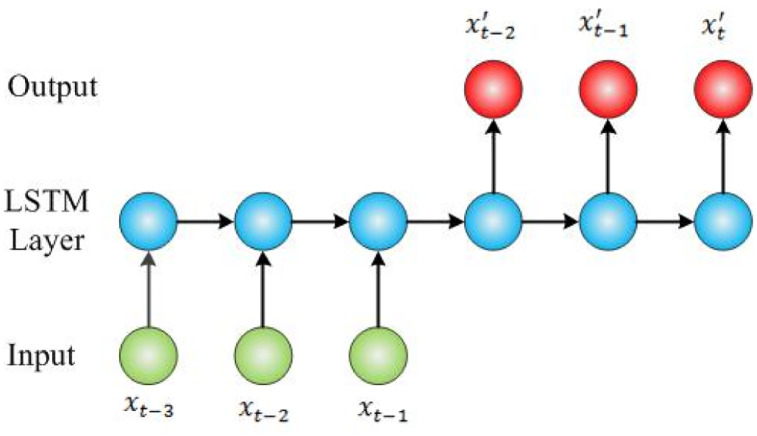
Structure of LSTM model.

LSTM can be used for sequence prediction, sequence classification, sequence generation, sequence to sequence prediction. In this work, we model hepatitis E prediction by sequence to sequence prediction. Two key factors of LSTM modeling are feature and time step. We take monthly incidence or monthly cases number as feature. Time step is set 3, which means that we predict next monthly incidence of hepatitis E, by using the previous three monthly data. The form of input and output is shown in Fig 3. Then, we choose $x_{t}^{{}^{\prime }}$ as the value of prediction. In this model, the number of nodes in the input, hidden and output layer are set 1, 4, 1, respectively.

For data preprocessing, we also normalized the raw data to [0, 1], as SVM method.

#### Parameters setting of LSTM

The data set of hepatitis E incidence only has 156 elements, since the model is prone to over-fitting. In order to overcome the above problem, we adopt dropout and regularization strategy. Dropout parameter between hidden layer and output layer is set to 0.15. Regularization parameter is set to 0.001. The iteration of training is set to 220. In addition, Optimization algorithm is the heart of machine learning, which affects the convergence and optimization of the algorithm. We also find that the Adam is faster and better than stochastic gradient descent (SGD) optimization method.

### Comparison metrics

In order to fairly compare the performance of the three models, we apply three commonly used quality indexes, including Root Mean Square Error (RMSE), Mean Absolute Percent Error (MAPE), Mean Absolute Error (MAE). RMSE is used to measure the discreteness of a group of numbers themselves, as shown in the formula 1. RMSE tends to be dominated by larger values. MAPE is widely used to measure the quality of a prediction model, as shown in the formula 2. The smaller the MAPE value is, the better the accuracy of the prediction model can be. MAE shows the actual prediction error, as shown in the formula 3.

$$\begin{array}{c} RMSE=\sqrt{\frac{1}{n}\sum_{i=1}^{N}{\left(y_{i}-{\hat{y}}_{i}\right)}^{2}} \end{array}$$ (1)

$$\begin{array}{c} MAPE=\sum_{i=1}^{N}\left|\frac{y_{i}-{\hat{y}}_{i}}{y_{i}}\right|\times \frac{100}{N} \end{array}$$ (2)

$$\begin{array}{c} MAE=\frac{1}{N}\sum_{i=1}^{N}\left|y_{i}-{\hat{y}}_{i}\right| \end{array}$$ (3)

## Results and discussion

### ARIMA model

For ARIMA model, we firstly observed the monthly incidence and cases number of hepatitis E. As can be seen from Figs 1 and 2, there are wave peaks in the middle of the data, and the two sides are lower. In order to accurately evaluate the stationarity of data, we adopted ADF to analyze raw data and first-order difference data. The result of ADF test in the incidence of hepatitis E is shown in Table 1. And the result of ADF test in the cases number of hepatitis E is shown in Table 2. A t-Statistic of less than 1%-Statistic and a p-value close to 0 indicate stationary series. We can observe from Table 1 that t-Statistic value(-1.9193) is more than 1%-Statistic(-3.4776) in the raw data, and p-value(0.3230) is far greater than 0. Hence, we need to use difference to make the data stationary. After first-order difference, t-Statistic(-3.5082) and p-value(0.0077) are suitable for stationary requirements. From Table 2, we can see that monthly cases number of hepatitis E and monthly incidence are consistent. After first-order difference, it can meet the requirement of stationarity.

**Table 1:** ADF test of monthly incidence of hepatitis E.

	t-Statistic	1%-Statistic	5%-Statistic	10%-Statistic	p-value
*d* = 0	-1.9193	-3.4776	-2.8822	-2.5778	0.3230
*d* = 1	-3.5082	-3.4776	-2.8822	-2.5778	0.0077

*d* = 0 denotes the raw data which is not processed by difference. *d* = 1 denotes the data which is processed by first-order difference.

**Table 2:** ADF test of monthly cases number of hepatitis E.

	t-Statistic	1%-Statistic	5%-Statistic	10%-Statistic	p-value
*d* = 0	-1.9195	-3.4776	-2.8822	-2.5778	0.3229
*d* = 1	-3.4968	-3.4776	-2.8822	-2.5778	0.0080

*d* = 0 denotes the raw data which is not processed by difference. *d* = 1 denotes the data which is processed by first-order difference.

After difference processing, we need to determine *p* of autoregressive(AR) model and *q* of moving average(MA) model. Grid-search of optimization was used to determine *p* and *q* by BIC criterion. For monthly incidence prediction, *p* and *q* ranged from 0 to 2. BIC value is shown in Table 3. We can observed that BIC value(-476.56) is the lowest when *p*, *q* were set to 1. That means we choose ARIMA(1,1,1) to predict incidence of hepatitis E. For monthly cases number prediction, *p* and *q* changed from 0 to 3. Table 4 showed the results of BIC by *p*, *q* ranging from 0 to 3. BIC value obtained the lowest value(1217.11) when *p* was set to 3 and *q* was set to 2. Finally, ARIMA(3,1,2) was determined to predict cases number of hepatitis E.

**Table 3:** BIC test for monthly incidence of hepatitis E by *p*, *q*.

	*q* = 0	*q* = 1	*q* = 2
*p* = 0	-466.59	-461.94	-461.02
*p* = 1	-461.98	-476.56	-472.98
*p* = 2	-459.45	-474.42	-472.85

*p* denotes the orders of AR model. *q* denotes the orders of MA model.

**Table 4:** BIC test for monthly cases number of hepatitis E by *p*, *q*.

	*q* = 0	*q* = 1	*q* = 2	*q* = 3
*p* = 0	1241.74	1246.53	1247.64	1228.27
*p* = 1	1246.52	1232.46	1235.61	1227.22
*p* = 2	1249.31	1233.72	1237.45	1225.63
*p* = 3	1248.11	1217.55	1217.11	1221.54

*p* denotes the orders of AR model. *q* denotes the orders of MA model.

### SVM model

**Fig 4:**
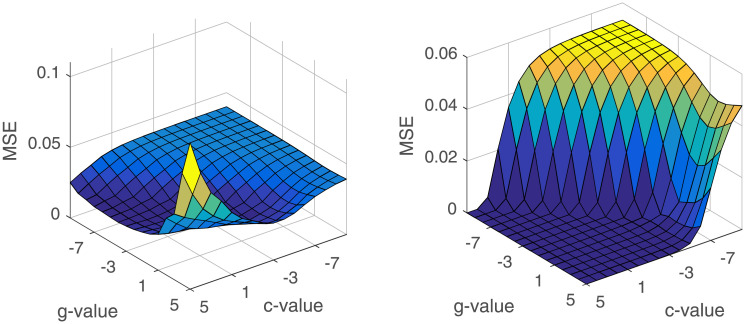
The results of grid search for *C* and *g* in SVM model. The left part is the result of SVM model for monthly incidence prediction. And the right part is the result of SVM model for monthly cases number prediction.

SVM model was implemented by drawing support from libSVM. As stated in above Methods section, we need to choose the best parameters to determine a SVM model. We adopted cross-validation and grid search to optimize penalty *C* and kernel function parameter *g*, which range from 2^−10^ to 2^5^. At this stage, MSE is the metrics to measure SVM model. The results of grid search was shown in Fig 4. The left part which is the result of incidence prediction, showed the change of MSE in the SVM model according different *C* and *g*. And the scales on x-axis and y-axis are the exponent of 2 in *C* and *g*. The right part is the result from monthly cases number prediction. Finally, for monthly incidence prediction, we set *C* and *g* with 8, 0.125, respectively. For monthly cases number prediction, we set *C* and *g* with 22, 0.01, in the SVM model.

### LSTM model

**Fig 5:**
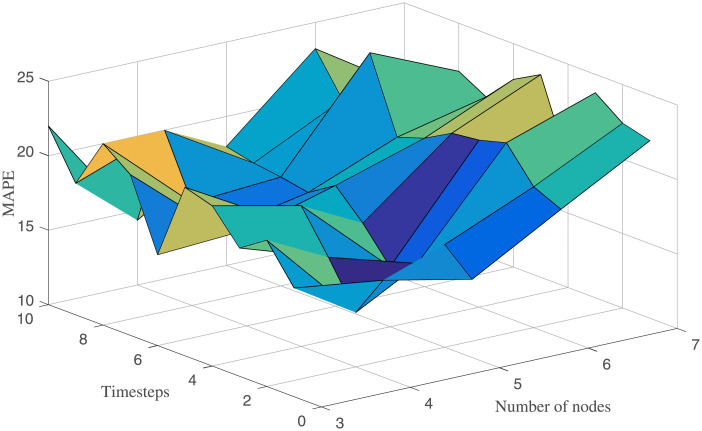
Influence of the nodes number and timestep on performance of LSTM model.

With regard to LSTM model, network structure need to be determined firstly. According to the above modeling method in the Methods part, we mainly study the influence of the nodes number and timestep on the performance of LSTM model. The result of grid search in the LSTM model is shown in Fig 5. The nodes number changes from 3 to 7, and the time step ranges from 0 to 10. We can observe from Fig 5 that the MAPE increases gradually, with the increase of the nodes number. Finally, we determined the LSTM model with 4 nodes and 3 time steps.

**Fig 6:**
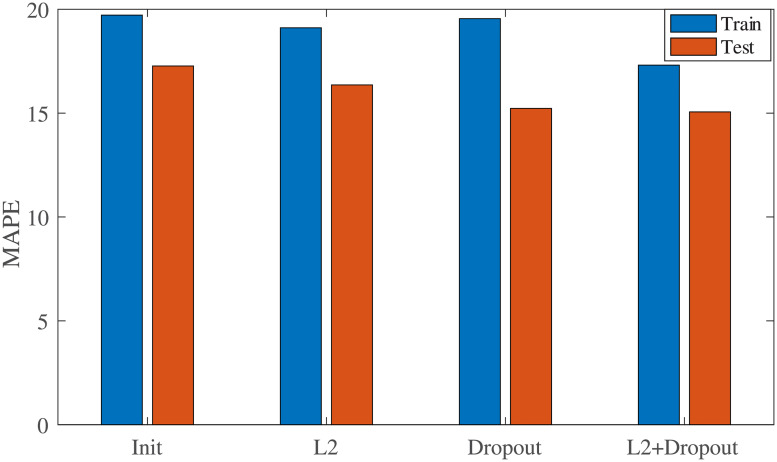
Influence of dropout and L2 on performance of LSTM model.

To overcome the problem of overfitting caused by limited training samples, we adopted dropout and regularization strategies in our LSTM model. Herein, we perform four sets of experiments: LSTM without dropout and L2, LSTM with only L2, LSTM with only dropout, and LSTM with L2 and dropout. Fig 6 shows the MAPE of the training and test sets. We can see from Fig 6, that LSTM with L2 and dropout has the best result on test set.

### Model comparison

**Fig 7:**
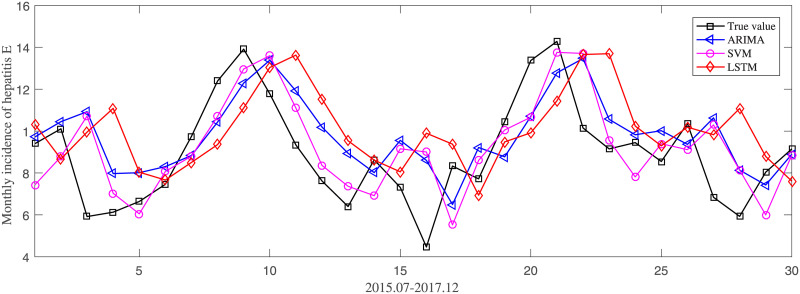
Comparison of different models for monthly incidence prediction.

**Fig 8:**
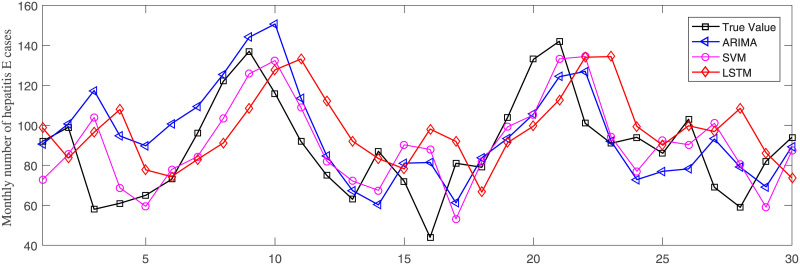
Comparison of different models for monthly cases number prediction.

ARIMA, SVM and LSTM were adopted to predict the monthly incidence of hepatitis E from July 2015 to December 2017. The prediction performance of each models was evaluated by three metrics, including RMSE, MAPE and MAE. For monthly incidence prediction, the detailed results of three metrics were shown in Table 5. Fig 7 illustrated the actual incidence curve and predicting curves of three models on MAPE. Similarly, we obtained the detailed results for monthly cases number prediction, as shown in Table 6. And Fig 8 illustrated the curves of monthly cases number of hepatitis E.

**Table 5:** Results of different models for monthly incidence prediction.

*Method*	RMSE	MAPE(%)	MAE
*ARIMA*	0.022	23.5	0.018
*SVM*	0.0204	21.70	0.0167
*LSTM*	0.01	15.08	0.011

**Table 6:** Results of different models for monthly cases number prediction.

*Method*	RMSE	MAPE(%)	MAE
*ARIMA*	22.25	23.6	18.003
*SVM*	20.0368	21.44	16.5815
*LSTM*	11.75	13.60	9.984

### Discussion

From Figs 1 and 2 we can see, that monthly incidence and cases number of hepatitis E have the same trend. The monthly incidence of hepatitis E had risen up slightly from 2009 to 2014, and declined from 2015.

ARIMA is a classic statistical prediction method, and also a typical representative of linear model. SVM is a representative model of traditional machine learning, which has good performance in classification and regression. LSTM is a deep learning model, which is most suitable for nonlinear regression. Therefore, we choose these three models for comparison, to find out the model suitable for the incidence prediction of hepatitis E. Comparing the three models in Fig 7, we observed that the trends of RMSE, MAPE and Mae are consistent. ARIMA got the worst performance on RMSE, MAPE and MAE. SVM is slightly better than ARIMA on all metrics. While, LSTM outperforms ARIMA and SVM significantly. Take MAPE for example, LSTM obtained a result of 15.08%, which drops about 6.6% and 8.4%, respectively comparing SVM and ARIMA. Observing the curves in Fig 8, we can see that ARIMA can capture the trend of hepatitis E, but the prediction deviation is large. This may be because ARIMA is a linear model and has insufficient learning ability for nonlinear parts. Nonlinear models, SVM and LSTM, have better performance than ARIMA.

For monthly cases number prediction, we got the same conclusion with monthly incidence prediction, shown in Table 6. LSTM outperforms SVM and ARIMA. Although the monthly incidence and cases number have the same trend, the order of magnitude is different. Accordingly, parameters of models and results of models are different. RMSE and MAE of monthly cases number is about 1000 times that of the monthly incidence. For ARIMA and SVM, MAPE is almost the same in two experiments. However, LSTM got a better MAPE 13.6%, which drop about 1.4% than that of monthly incidence. In general, machine learning models make great sense in decision making and were shown useful in the prediction of monthly incidence and cases number in hepatitis E. And LSTM model is much better than ARIMA and SVM.

## Conclusion

In this work, we adopted ARIMA, SVM, and LSTM to predict monthly incidence and cases number of hepatitis E in Shandong Province, China. To verify the effectiveness of the methods, RMSE, MAPE and MSE metrics were applied to evaluate the performances of each model. According to the experimental results, we can draw the following conclusions. Linear model(ARIMA) is inferior to nonlinear modes(SVM, LSTM). LSTM we proposed has competitive performance of predicting monthly incidence of hepatitis E.

## Supporting information

S1 Data (XLSX)

S2 Data (XLSX)
